# Supplementary material for: cGAS-STING signalling regulates microglial chemotaxis in genome instability
Source: Nucleic Acids Res. 2023 Dec 12;52(3):1188–206. doi: 10.1093/nar/gkad1184 (PMC10853792; doi:10.1093/nar/gkad1184)
Supplement: gkad1184_supplemental_file [file gkad1184_supplemental_file.pdf]

Supplementary information

cGAS-STING signalling regulates microglial chemotaxis in genome instability

Emily J. Talbot, Lisha Joshi, Peter Thornton, Mahya Dezfouli, Kalliopi Tsafou,
Michael Perkinton and Svetlana V. Khoronenkova*

* Corresponding author: sk870@cam.ac.uk

Supplementary Table 1: Cell lines, reagents and software programs used in this work.

Supplementary Table 2: Human tissue sample information.

Supplementary Table 3: Differential chemokine gene expression analysis.

Supplementary Figure 1: ATM deficiency activates the cGAS-STING pathway in human microglia-like cells.

Supplementary Figure 2: Cytosolic DNA in ATM deficiency primarily derives from micronuclei.

Supplementary Figure 3: Inhibiting mitotic entry attenuates STING pathway activation following loss of ATM function.

Supplementary Figure 4: STING drives expression and secretion of type I interferons following ATM inhibition in microglia-like cells.

Supplementary Figure 5: Persistent DNA damage drives STING-dependent chemokine induction in microglia-like cells.

Supplementary references.

Supplementary Table 1. Cell lines, reagents and software programs used in this work.

REAGENT or RESOURCE	SOURCE	IDENTIFIER
**Experimental Models: Cell Lines**		
HMC3 cells	Kind gift from Dr Brian Bigger (University of Manchester, UK)	[1]
STING1 knockout HMC3 cells	This paper	
C20 cells	Kind gift from Dr David Alvarez-Carbonell (Case Western Reserve University)	[2]
ATM knockout C20 cells	Khoronenkova Laboratory	[3]
iCell® Microglia	FujiFilm CDI	R1131
**Bacterial Strains**		
DH5α	Invitrogen	18265017
NEB® 10-beta	New England Biolabs	C3019
**Recombinant DNA**		
pSpCas9(BB)-2A-Puro (PX459) V2.0	Kind gift from Feng Zhang	Addgene plasmid #62988; http://n2t.net/addgene:62988; RRID:Addgene, 62988[4]
**Oligonucleotides**	**Sequence (5' → 3')**	**SOURCE**
**Cloning**		
STING sgRNA sense	caccgGCTGGGACTGCTGTTAAACG	This paper
STING1 sgRNA anti-sense	aaacCGTTTAACAGCAGTCCCAGCc	This paper
**Fragment analysis**		
STING1-exon 3 Forward	GCTAGGCATCAAGGGAGTGA	This paper
STING1-exon 3 Reverse	TGGATTTCTTGGTGCCCACA	This paper
**RT-qPCR**		
CCL5 Forward	GCTGTCATCCTCATTGCTACTG	[5]
CCL5 Reverse	TGGTGTAGAAATACTCCTTGATGTG	
CGAS Forward	CTGAACACCGGGAGCTACTA	This paper
CGAS Reverse	TTGAATTCTGGGGACTTCCAGT	

CXCL10 Forward	CCATTCTGATTTGCTGCCTTATC	[6]
CXCL10 Reverse	TACTAATGCTGATGCAGGTACAG	
IFIT2 Forward	CTGCAACCATGAGTGAGAACAA	This paper
IFIT2 Reverse	CTCCCTCCATCAAGTTCCAGG	
IFNB1 Forward	ATGACCAACAAGTGTCTCCTCC	[7]
IFNB1 Reverse	GCTCATGGAAAGAGCTGTAGTG	
IL6 Forward	AGACAGCCACTCACCTCTTCAG	[3]
IL6 Reverse	TTCTGCCAGTGCCTCTTTGCTG	
STING1 Forward	GGGCTGGCATGGTCATATTA	[8]
STING1 Reverse	TACTCAGGTTATCAGGCACC	
TFAM Forward	GCGCTCCCCCTTCAGTTTTG	[9]
TFAM Reverse	GTTTTTGCATCTGGGTTCTGAGC	
RPS13 Forward	CGAAAGCATCTTGAGAGGAACA	[3]
RPS13 Reverse	TCGAGCCAAACGGTGAATC	
IPO8 Forward	GGCACCACTCAGCGAGGAT	[10]
IPO8 Reverse	TGTTGTTCAATCTTCTTCTTTGCCT	
**siRNA Oligonucleotides**		
siCtrl (negative control)	GAUCGAUUCGCAGUUGAUUCU	This paper
sicGAS (SMART Pool)	Dharmacon siGENOME	M-015607-01
siSTING	GGCAUCAAGGAUCGGGUUU	[11]
**Antibodies** (WB = western blotting; IF = immunofluorescence; FC = flow cytometry)		
β-Actin (WB)	Abcam	ab6276
ATM (WB)	Abcam	ab78
ATM (WB)	Bethyl Laboratories	A300-299A
ATM pSer1981 (WB)	Abcam	ab81292
BrdU (FC)	BD Biosciences	347580
cGAS (WB, IF)	Cell Signaling	15102
CHK2 (WB)	Cell Signaling	3440
CHK2 pThr68 (WB)	Cell Signaling	2661
Cleaved caspase-3 (WB, IF)	Cell Signaling	9664
Fibrillarin (WB)	Abcam	ab4566
GAPDH (WB)	Cell Signaling	2118

GAPDH (WB)	Proteintech	60004-1-Ig
γH2AX (WB)	Millipore	05-636
IRF3 (WB)	Santa Cruz	sc-33641
IRF3 (IF)	Cell Signaling	11904
IRF3 pSer396 (WB)	Cell Signaling	4947
POLR2A (WB)	Covance	MMS-128P
STAT1 (WB)	Cell Signaling	9176
STAT1 pTyr701 (WB)	Cell Signaling	9167
STING (WB)	Cell Signaling	13647
STING (IF)	Abcam	ab181125
STING pSer366 (WB)	Cell Signaling	19781
TBK1 (WB)	Abcam	ab40676
TBK1 pSer172 (WB)	Cell Signaling	5483
α/β-Tubulin (WB)	Cell Signaling	2148
Vinculin (WB, IF)	Abcam	ab18058
Vinculin (WB)	Proteintech	26520-1-AP
AlexaFluor 680 goat anti-rabbit (WB)	Molecular Probes	A27042
IR Dye 800 goat anti-mouse (WB)	LI-COR Biosciences	926-32210
AlexaFluor 488 goat anti-mouse (IF, FC)	Invitrogen	A11001
AlexaFluor 488 goat anti-rabbit (IF)	Invitrogen	A32731
AlexaFluor 594 goat anti-mouse (IF)	Invitrogen	A-11032
Alexa Fluor 594 goat anti-rabbit (IF)	Abcam	ab150080
**Chemicals, enzymes, and other reagents**		
Aphidicolin	Merck	178273
AZD1390 (ATM inhibitor)	Selleckchem	S8680
5-Bromodeoxyuridine (BrdU)	Merck	B9285
Camptothecin	Cambridge Bioscience	CAY11694
RO-3306 (CDK1 inhibitor)	Tocris Bioscience	4181
5,6-Dichloro-1-β-ribofuranosylbenzimidazole (DRB)	Cayman Chemical Company	10010302

Etoposide	Cambridge Bioscience	CAY12092
Mitomycin C	Merck	M0503
N-acetyl-L-cysteine (NAC)	Merck	A9165
STING agonist diABZI	Selleckchem	S8796
Puromycin	Santa Cruz	sc-108071
4,5-Dihydroxy-1,3-benzenedisulfonic acid (tiron)	Merck	172553
Triptolide	Cayman Chemical Company	11973
α-Amanitin	Cayman Chemical Company	17898
Catalase	Merck	C1345
Recombinant human IFN-β	R&D Systems	8499-IF
Phusion High-Fidelity DNA Polymerase	ThermoFisher	F530
Lipofectamine RNAiMax	Invitrogen	13778-150
DNase I	ThermoFisher	EN0521
2-well silicone inserts	Ibidi	80209
TRI Reagent®	Merck	T9424
**Kits and assays**		
Glial-Mag Magnetofection	OzBiosciences	GL00500
KAPA mRNA HyperPrep Kit	Roche	08098115702
NGS Fragment Kit (1-6,000 bp)	Agilent Technologies	DNF-473
RNeasy Mini Kit	QIAGEN	74106
Agilent RNA 6000 Pico Kit	Agilent Technologies	5067-1513
RNAdvance Cell v2	Beckman Coulter	A47943
qPCRBIO cDNA Synthesis Kit	PCR Biosystems	PB30.11-10
qPCRBIO SyGreen Blue Mix Lo-ROX	PCR Biosystems	PB20.15-20
MSD U-PLEX human cytokine assay	Meso Scale Discovery	K151AEL-2
**Software and Algorithms**		
CellProfiler v4.0.7	Broad Institute	
FlowJo v10.4.2	FlowJo	
GraphPad Prism v9.4.1	GraphPad Software	

Image Studio™ Lite Software v5.2	LI-COR
Image J	NIH
DISCOVERY WORKBENCH v4.0	Meso Scale Discovery
QuantStudio™ Design & Analysis v1.5.1	ThermoFisher

Supplementary Table 2. Human tissue sample information (source: NIH Neurobiobank, University of Maryland, United States). PMI = post-mortem interval, RIN = RNA integrity number, NA = not available.

ID	Diagnosis	Region	Brain Section	Age (years)	Sex	PMI (hours)	RIN	Cause of death
1259	Control	Cerebellum	Vermis	24	Male	8	9.2	Accident MVA
6018	A-T	Cerebellum	Vermis	24	Male	9	8.1	Complication of Disorder
5351	Control	Cerebellum	Vermis	44	Male	22	7	Cardiac Arrhythmia
5748	A-T	Cerebellum	Vermis	44	Male	29	NA	Lymphoma / T Cell acute lymphoblastic leukemia
6062	Control	Cerebellum	Vermis	32	Female	8	6	Mixed Drug Intoxication
6249	A-T	Cerebellum	Vermis	31	Female	4	3.2	Complications of disorder

Supplementary Table 3. Differential chemokine expression analysis. The data for chemokine genes as in Figure 4D are shown (IFN-β-treated *vs* vehicle). Genes that are not significantly differentially expressed are highlighted in grey (adjusted p-value > 0.05, adj.P.Val). logFC = log2FC, AveExpr = average log-expression, t = moderated t-statistic.

**Comparison**	**symbol**	**gene_name**	**logFC**	**AveExpr**	**t**	**P.Value**	**adj.P.Val**
IFN-β *vs* vehicle, 6 h	CCL2	chemokine (C-C motif) ligand 2	3.585471	7.604373	5.529698	4.19E-05	0.000192
IFN-β *vs* vehicle, 24 h	CCL2	chemokine (C-C motif) ligand 2	3.724959	7.604373	4.228267	0.000609	0.00283
IFN-β *vs* vehicle, 6 h	CCL3	chemokine (C-C motif) ligand 3	2.035604	2.315967	8.963174	1.15E-08	1.21E-07
IFN-β *vs* vehicle, 24 h	CCL3	chemokine (C-C motif) ligand 3	0.559293	2.315967	1.972764	0.061679	0.12522
IFN-β *vs* vehicle, 6 h	CCL3L1	chemokine (C-C motif) ligand 3-like 1	2.924948	3.265784	9.603479	8.92E-09	9.61E-08
IFN-β *vs* vehicle, 24 h	CCL3L1	chemokine (C-C motif) ligand 3-like 1	0.802923	3.265784	2.020491	0.05747	0.118549
IFN-β *vs* vehicle, 6 h	CCL4	chemokine (C-C motif) ligand 4	2.47947	3.939134	8.835658	3.64E-08	3.42E-07
IFN-β *vs* vehicle, 24 h	CCL4	chemokine (C-C motif) ligand 4	0.231201	3.939134	0.619044	0.543221	0.661649
IFN-β *vs* vehicle, 6 h	CCL5	chemokine (C-C motif) ligand 5	1.61142	1.333714	3.287189	0.003737	0.009867
IFN-β *vs* vehicle, 24 h	CCL5	chemokine (C-C motif) ligand 5	1.5179	1.333714	3.13533	0.005276	0.017068
IFN-β *vs* vehicle, 6 h	CCL7	chemokine (C-C motif) ligand 7	5.646911	4.775432	5.374496	5.71E-05	0.000252
IFN-β *vs* vehicle, 24 h	CCL7	chemokine (C-C motif) ligand 7	5.569377	4.775432	3.883858	0.001266	0.005236
IFN-β *vs* vehicle, 6 h	CCL8	chemokine (C-C motif) ligand 8	9.90975	2.005463	7.206258	1.6E-06	1.02E-05
IFN-β *vs* vehicle, 24 h	CCL8	chemokine (C-C motif) ligand 8	11.57748	2.005463	4.565436	0.000284	0.001502
IFN-β *vs* vehicle, 6 h	CCL13	chemokine (C-C motif) ligand 13	4.975606	-0.74491	10.98405	3.2E-10	4.81E-09
IFN-β *vs* vehicle, 24 h	CCL13	chemokine (C-C motif) ligand 13	4.759297	-0.74491	6.896886	7.66E-07	9.13E-06
IFN-β *vs* vehicle, 6 h	CXCL8	chemokine (C-X-C motif) ligand 8	-1.39268	7.074233	-4.80979	0.000154	0.0006
IFN-β *vs* vehicle, 24 h	CXCL8	chemokine (C-X-C motif) ligand 8	-1.33479	7.074233	-3.79725	0.001391	0.00567
IFN-β *vs* vehicle, 6 h	CXCL9	chemokine (C-X-C motif) ligand 9	12.49648	-1.59857	25.50445	2.04E-17	2.76E-15
IFN-β *vs* vehicle, 24 h	CXCL9	chemokine (C-X-C motif) ligand 9	6.715951	-1.59857	10.77883	4.51E-10	1.31E-08
IFN-β *vs* vehicle, 6 h	CXCL10	chemokine (C-X-C motif) ligand 10	14.61706	1.487149	6.135142	1.1E-05	5.79E-05
IFN-β *vs* vehicle, 24 h	CXCL10	chemokine (C-X-C motif) ligand 10	12.6942	1.487149	5.559405	3.46E-05	0.000246
IFN-β *vs* vehicle, 6 h	CXCL11	chemokine (C-X-C motif) ligand 11	12.35785	-1.24895	19.23714	2.37E-14	1.02E-12
IFN-β *vs* vehicle, 24 h	CXCL11	chemokine (C-X-C motif) ligand 11	8.843962	-1.24895	12.17954	1.07E-10	3.75E-09
IFN-β *vs* vehicle, 6 h	CX3CL1	chemokine (C-X3-C motif) ligand 1	-2.29832	-2.04436	-2.86734	0.009153	0.021374
IFN-β *vs* vehicle, 24 h	CX3CL1	chemokine (C-X3-C motif) ligand 1	-3.5532	-2.04436	-5.39112	2.31E-05	0.000175

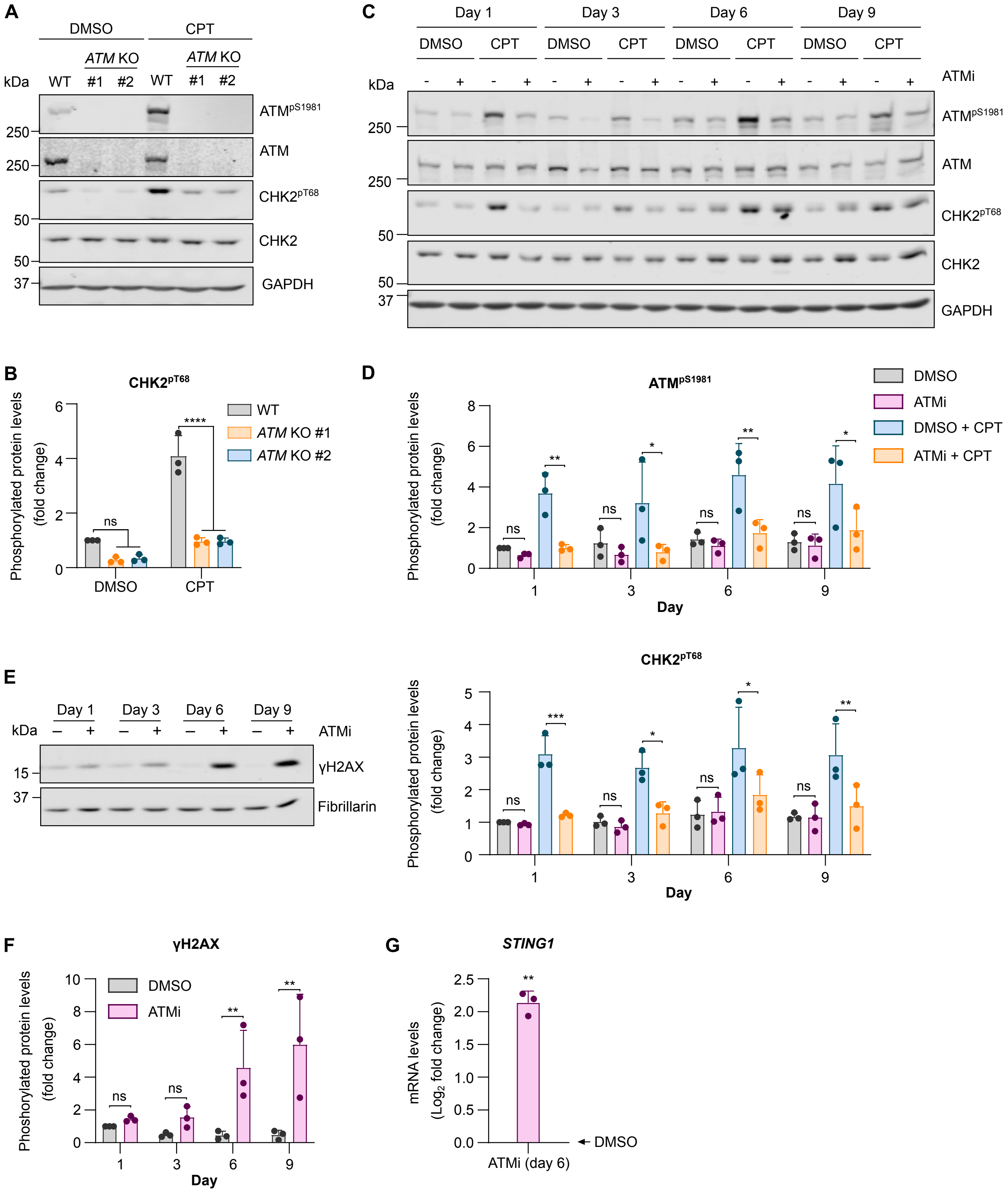

Talbot *et al.*, Supplementary Figure 1

Supplementary Figure 1: ATM deficiency activates the cGAS-STING pathway in human microglia-like cells.

(A) Representative immunoblot of *ATM* KO C20 microglial cells (clones #1 and #2) treated with 1 µM camptothecin (CPT), or DMSO as a control, for 1 h. Loading control: GAPDH.
(B) Relative quantification of $CHK2^{pT68}$ normalised to total CHK2 levels in C20 cells as in (A). Mean fold change ± SD (n=3). Two-way ANOVA with Tukey's post-hoc comparison test.
(C) Representative immunoblot of HMC3 cells treated as in Figure 1C. CPT or DMSO treatment as in (A). Loading control: GAPDH.
(D) Quantification of ATM^{pS1981} and $CHK2^{pT68}$ normalised to respective total protein levels as in (C). Data are normalised to day 1 DMSO. Mean fold change ± SD (n=3). Two-way ANOVA with Tukey's post-hoc comparison test.
(E) Representative immunoblot of nuclear extracts of HMC3 cells treated as in (C). Loading control: Fibrillarin.
(F) Quantification of γH2AX normalised to Fibrillarin levels as in (E). Data are normalised to day 1 DMSO. Mean fold change ± SD (n=3). Two-way ANOVA with Sidak's multiple comparisons test.
(G) Relative *STING1* mRNA levels in HMC3 cells treated with ATMi for 6 days. C_q values normalised to *RPS13* and DMSO-treated cells (indicated by arrow). Mean log_2 fold change ± SD (n=3). One-sample *t*-test comparing to a hypothetical mean of 0.
*p ≤ 0.05; **p ≤ 0.01; ***p ≤ 0.001; ****p ≤ 0.0001; ns = not significant.

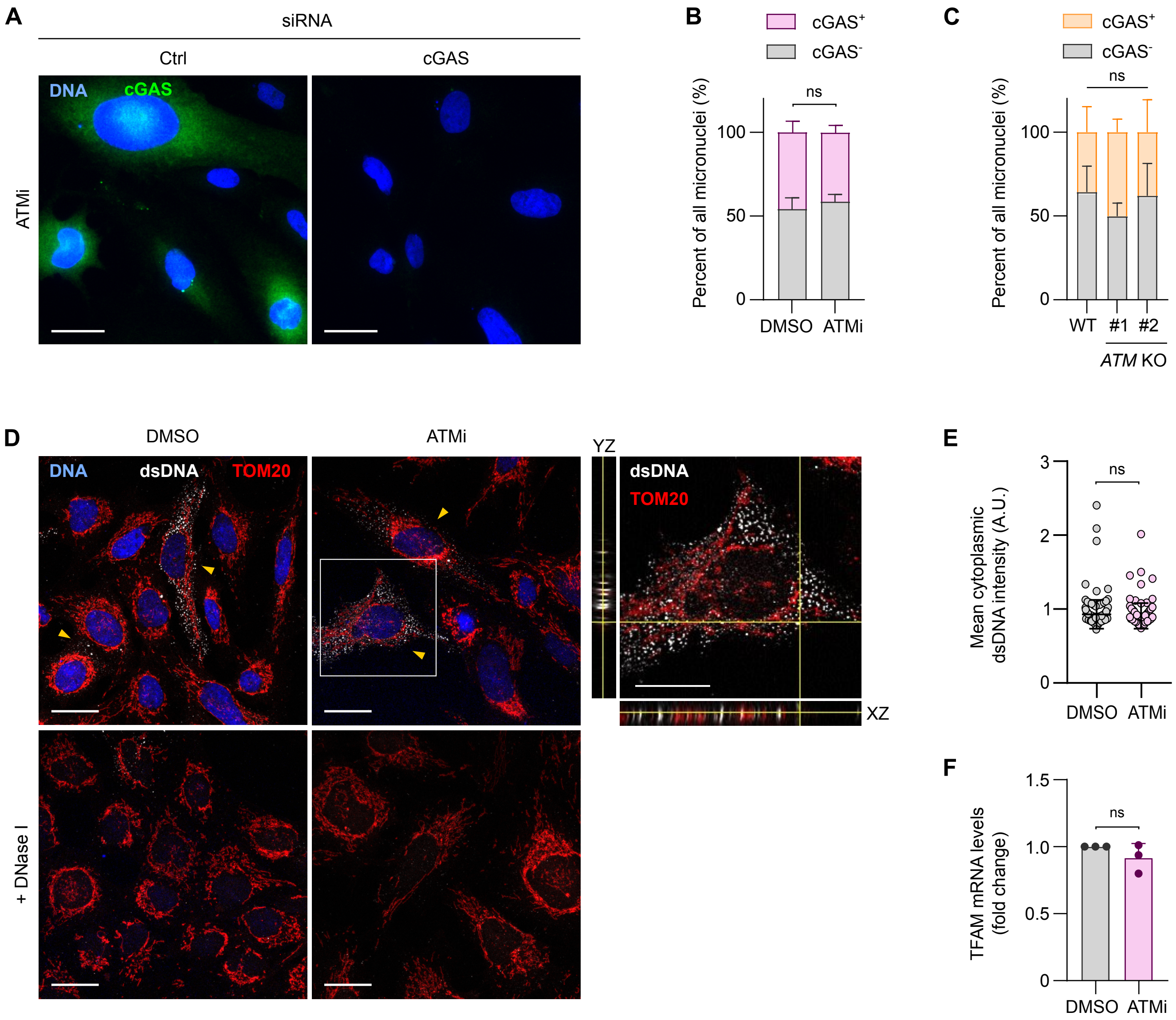

Talbot *et al.*, Supplementary Figure 2

Supplementary Figure 2. Cytosolic DNA in ATM deficiency primarily derives from micronuclei.

(A) **I**mmunofluorescence microscopy images of HMC3 cells treated with 10 nM AZD1390 (ATMi) for 6 days in combination with Ctrl or cGAS siRNA. 20x objective, scale bar = 40 μm.
(B) Percentage of total MN positive or negative for cGAS as in Figure 2G. Mean ± SD (n=3). Two-way ANOVA with Tukey's post-hoc comparison test.
(C) Percentage of total MN positive or negative for cGAS as in Figure 2H. Mean ± SD (n=4). Two-way ANOVA with Tukey's post-hoc comparison test.
(D) Confocal microscopy images of HMC3 cells treated with 10 nM AZD1390 (ATMi), or DMSO as a control, for 6 days. Blue: DNA (DAPI), grey: dsDNA, red: TOM20 (mitochondria). Maximum intensity projections from Z-stacks, 40x oil immersion objective, scale bar = 15 μm. Yellow arrows indicate cells with cytosolic dsDNA. Inset: single Z-plane of highlighted ATMi-treated cell with YZ and XZ projections. Representative of 2 independent experiments.
(E) Quantification of cytoplasmic dsDNA intensity per cell as in (D). Mean intensity ± SD (n=1, at least 100 cells analysed per condition). Unpaired, two-tailed Student's *t*-test. A.U. = arbitrary units.
(F) Relative *TFAM* mRNA levels in HMC3 cells treated with ATMi or DMSO for 6 days. C_q values normalised to *IPO8* and DMSO-treated cells. Mean log_2 fold change ± SD (n=3). One-sample *t*-test, hypothetical mean of 1.
ns = not significant.

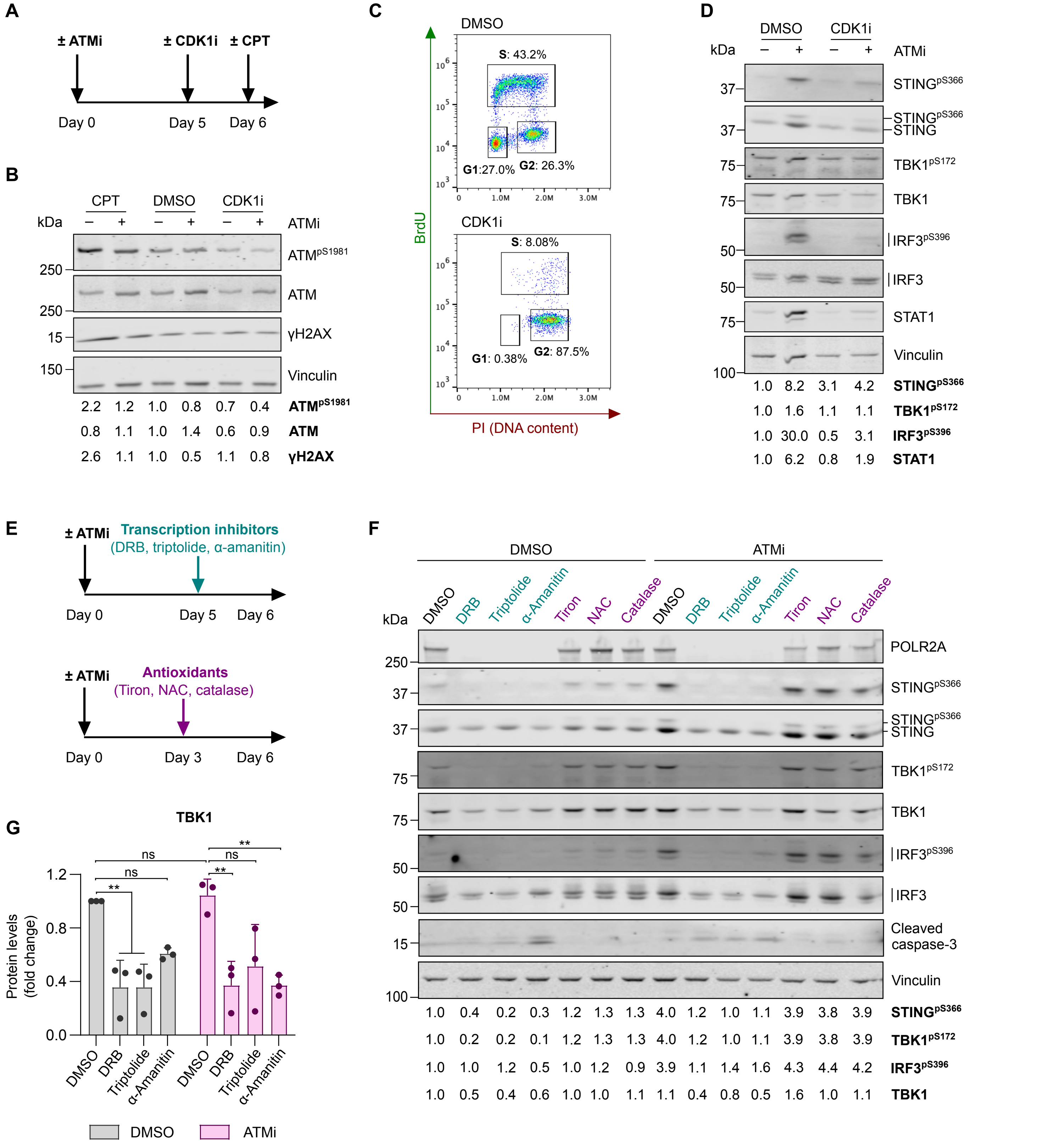

Talbot *et al.*, Supplementary Figure 3

Supplementary Figure 3: Inhibiting mitotic entry attenuates STING pathway activation following loss of ATM function.

(A) Schematic of workflow. HMC3 cells were treated with 10 nM ATMi, or DMSO as a control, for 6 days in the presence of 9 µM RO-3306 (CDK1i) or DMSO for the final 24 h. To confirm ATM inhibition, cells were treated with 1 µM camptothecin (CPT) or DMSO for 1 h.

(B) Representative immunoblot of HMC3 cells treated as in (A). Loading control: Vinculin. Quantification of phosphorylated proteins and ATM is relative to respective total proteins and vinculin, respectively, and normalised to DMSO control.

(C) Cells were treated as in (A), pulsed with 10 µM BrdU and analysed by BrdU-PI (propidium iodide) flow cytometry. Cell cycle distribution based on BrdU and PI intensity in DMSO (asynchronous) and CDK1i-treated cells (arrested in G2) in a representative experiment.

(D) Representative immunoblot of HMC3 cells treated with CDK1i, or DMSO as a control, as in (A). Loading control: Vinculin. Quantification of phosphorylated proteins and STAT1 is relative to respective total proteins and vinculin, respectively, and normalised to day 6 DMSO control.

(E) Schematic of workflow. HMC3 cells were treated with 10 nM ATMi, or DMSO as a control, for 6 days in the presence of 100 µM 5,6-dichloro-1-β-D-ribofuranosylbenzimidazole (DRB), 1 µM triptolide, or 2 µg/mL α-amanitin for the final 24 h. Alternatively, cells were treated for the final 72 h with 10 µM tiron, 5 µM N-acetyl-L-cysteine (NAC) or 100 U/mL catalase.

(F) Representative immunoblot of HMC3 cells treated as in (E). Loading control: Vinculin. Quantification of phosphorylated proteins and TBK1 is relative to respective total proteins and vinculin, respectively, and normalised to day 6 DMSO control.

(G) Quantification of TBK1 normalised to vinculin and Day 6 DMSO control in cells treated with transcription inhibitors as in (E-F). Mean fold change ± SD (n=3). Two-way ANOVA with Tukey's multiple comparisons test.

**$p \leq 0.01$; ns = not significant.

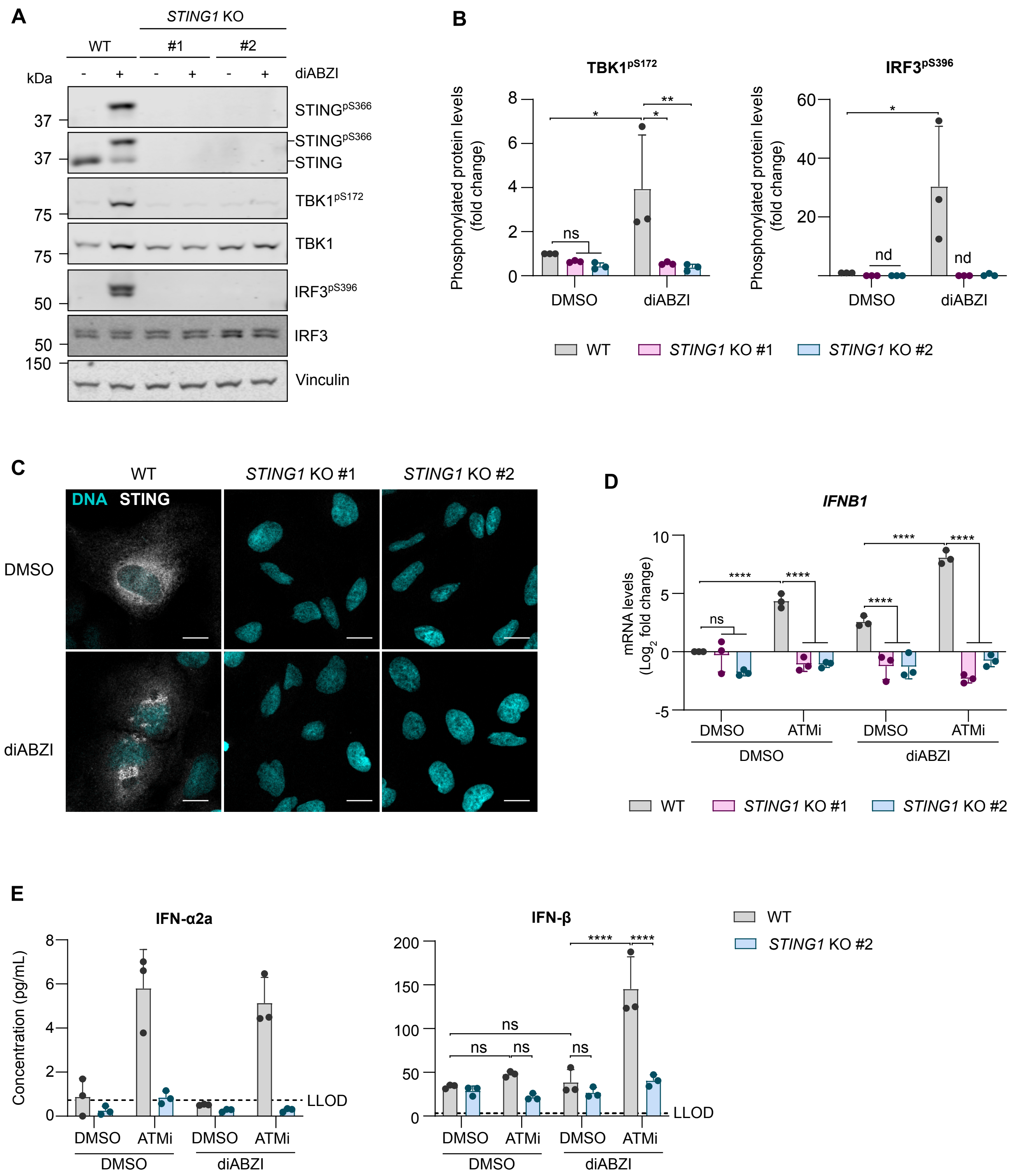

Talbot *et al.*, Supplementary Figure 4

Supplementary Figure 4. STING drives expression and secretion of type I interferons following ATM inhibition in microglia-like cells.

(A) Immunoblot of WT and *STING1* KO HMC3 cells (clones #1 and #2) treated with 1 μM diABZI, or DMSO as a control, for 2 h. Loading control: vinculin.

(B) Relative quantification of $\text{TBK1}^{\text{pS172}}$ and $\text{IRF3}^{\text{pS396}}$ as in (A) normalised to respective total protein and WT DMSO-treated cells. Mean ± SD (n=3). Nd: not detected. Two-way ANOVA with Tukey's post-hoc comparison test.

(C) Confocal microscopy images of cells as in (A). Cyan: DNA (DAPI), grey: STING. Single Z-plane image, 40x oil immersion objective, scale bar = 10 μm.

(D) Relative mRNA levels of *IFNB1* in WT and *STING1* KO HMC3 cells treated with 10 nM AZD1390 (ATMi), or DMSO as a control, for 6 days. Cells were stimulated with 1 μM diABZI, or DMSO as a control, for 5 h. C_q values normalised to *RPS13* and the DMSO-treated WT condition. Mean $\log_2$ fold change ± SD (n=3). Two-way ANOVA with Tukey's post-hoc comparison test.

(E) Concentration of interferons in cell culture supernatants. Mean ± SD (n=3). Dashed line indicates lower limit of detection (LLOD, pg/mL): IFN-α2a = 0.728, IFN-β = 3.28.

*p ≤ 0.05; **p ≤ 0.01; ****p ≤ 0.0001; ns = not significant.

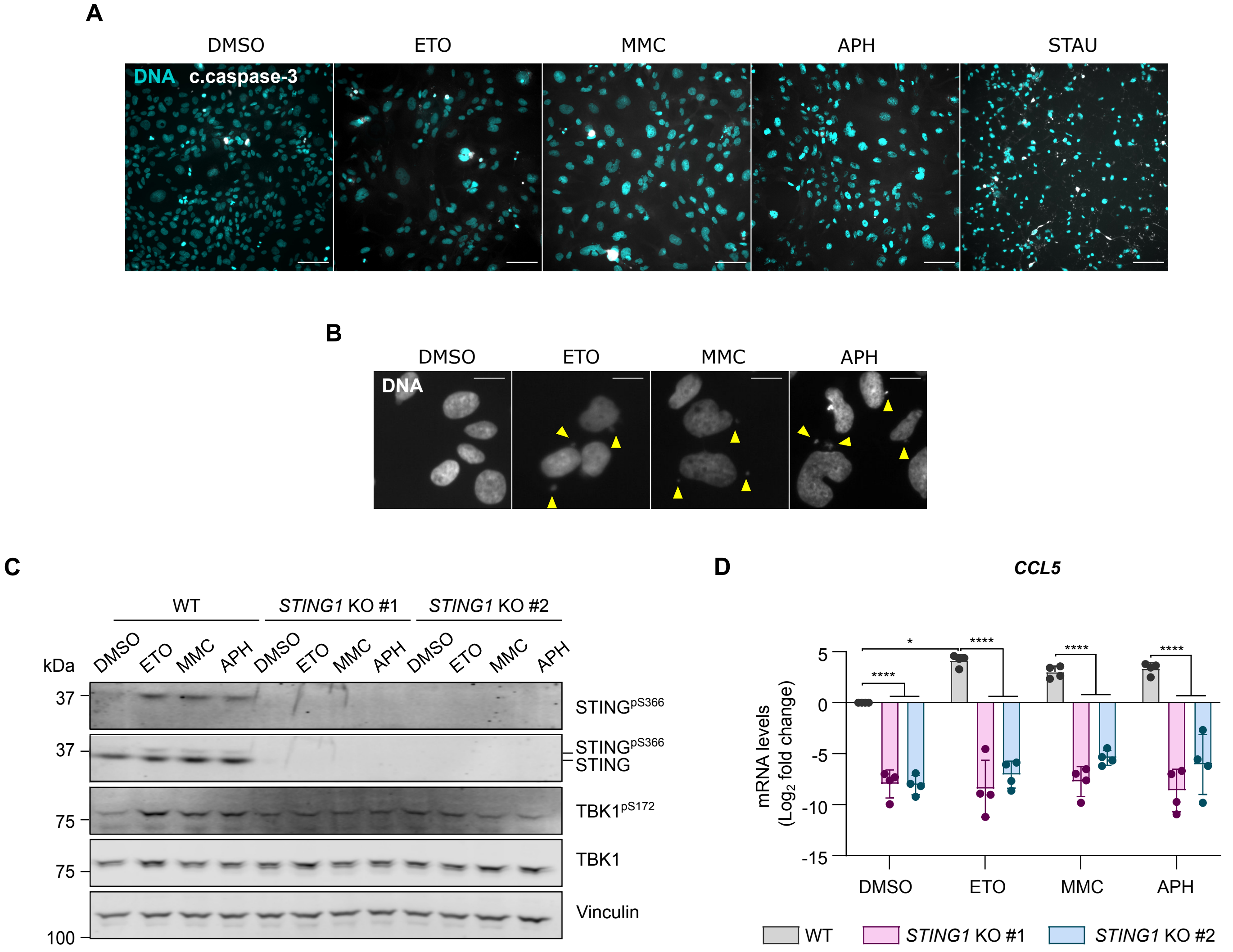

Talbot *et al.*, Supplementary Figure 5

Supplementary Figure 5. Persistent DNA damage drives STING-dependent chemokine induction in microglia-like cells.

(A) Immunofluorescence microscopy images of WT and *STING1* KO HMC3 cells treated with 250 nM etoposide (ETO), 30 nM mitomycin C (MMC), 150 nM aphidicolin (APH), or DMSO as a control, for 6 days. Treatment with 1 μM staurosporine for 6 h served as a positive control of apoptosis. Cyan: DNA (DAPI), grey: cleaved caspase-3 (c.caspase-3). 20x objective, scale bar = 100 μm.
(B) Representative immunofluorescence microscopy images demonstrating the formation of micronuclei. Grey: DNA (DAPI). 20x objective, scale bar = 20 μm.
(C) Representative immunoblot of cells treated as in (A). Loading control: Vinculin.
(D) Relative mRNA levels of *CCL5*. C_q values normalised to *RPS13* and the DMSO-treated WT condition. Mean $\log_2$ fold change ± SD (n=3). Two-way ANOVA with Tukey's post-hoc comparison test.
*p ≤ 0.05; ****p ≤ 0.0001.

SUPPLEMENTARY REFERENCES

1. Janabi, N., Peudenier, S., Héron, B., Ng, K.H., and Tardieu, M. (1995). Establishment of human microglial cell lines after transfection of primary cultures of embryonic microglial cells with the SV40 large T antigen. Neurosci. Lett. *195*, 105–108. 10.1016/0304-3940(94)11792-H.

2. Garcia-Mesa, Y., Jay, T.R., Checkley, M.A., Luttge, B., Dobrowolski, C., Valadkhan, S., Landreth, G.E., Karn, J., and Alvarez-Carbonell, D. (2017). Immortalization of primary microglia: a new platform to study HIV regulation in the central nervous system. J. Neurovirol. *23*, 47–66. 10.1007/s13365-016-0499-3.

3. Bourseguin, J., Cheng, W., Talbot, E., Hardy, L., Lai, J., Jeffries, A.M., Lodato, M.A., Lee, E.A., and Khoronenkova, S.V. (2022). Persistent DNA damage associated with ATM kinase deficiency promotes microglial dysfunction. Nucleic Acids Res. *50*, 2700–2718. 10.1093/nar/gkac104.

4. Ran, F.A., Hsu, P.D., Wright, J., Agarwala, V., Scott, D.A., and Zhang, F. (2013). Genome engineering using the CRISPR-Cas9 system. Nat. Protoc. *8*, 2281–2308. 10.1038/nprot.2013.143.

5. Jenkins, R.W., Clarke, C.J., Canals, D., Snider, A.J., Gault, C.R., Heffernan-Stroud, L., Wu, B.X., Simbari, F., Roddy, P., Kitatani, K., et al. (2011). Regulation of CC Ligand 5/RANTES by Acid Sphingomyelinase and Acid Ceramidase. J. Biol. Chem. *286*, 13292–13303. 10.1074/jbc.M110.163378.

6. Lama, L., Adura, C., Xie, W., Tomita, D., Kamei, T., Kuryavyi, V., Gogakos, T., Steinberg, J.I., Miller, M., Ramos-Espiritu, L., et al. (2019). Development of human cGAS-specific small-molecule inhibitors for repression of dsDNA-triggered interferon expression. Nat. Commun. *10*, 2261. 10.1038/s41467-019-08620-4.

7. Kim, J.-E., Kim, Y.-E., Stinski, M.F., Ahn, J.-H., and Song, Y.-J. (2017). Human Cytomegalovirus IE2 86 kDa Protein Induces STING Degradation and Inhibits cGAMP-Mediated IFN-β Induction. Front. Microbiol. *8*, 154.

8. Wang, Y.-Y., Jin, R., Zhou, G.-P., and Xu, H.-G. (2016). Mechanisms of transcriptional activation of the stimulator of interferon genes by transcription factors CREB and c-Myc. Oncotarget *7*, 85049–85057. 10.18632/oncotarget.13183.

9. Zhang, R., and Wang, J. (2018). HuR stabilizes TFAM mRNA in an ATM/p38-dependent manner in ionizing irradiated cancer cells. Cancer Sci. *109*, 2446–2457. 10.1111/cas.13657.

10. Jansen, A.H., Mamber, C., Kooijman, L., Orre, M., Koot, S., Dooves, S., Dimayuga Smith, V., Kamphuis, W., Hol, E.M., Kirk, C.J., et al. (2013). Reactive glia show increased immunoproteasome activity in Alzheimer’s disease. Brain *136*, 1415–1431. 10.1093/brain/awt083.

11. Herzner, A.-M., Hagmann, C.A., Goldeck, M., Wolter, S., Kübler, K., Wittmann, S., Gramberg, T., Andreeva, L., Hopfner, K.-P., Mertens, C., et al. (2015). Sequence-specific activation of the DNA sensor cGAS by Y-form DNA structures as found in primary HIV-1 cDNA. Nat. Immunol. *16*, 1025–1033. 10.1038/ni.3267.
